# Increases in absenteeism among health care workers in Hong Kong during influenza epidemics, 2004–2009

**DOI:** 10.1186/s12879-015-1316-y

**Published:** 2015-12-29

**Authors:** Dennis K. M. Ip, Eric H. Y. Lau, Yat Hung Tam, Hau Chi So, Benjamin J. Cowling, Henry K. H. Kwok

**Affiliations:** WHO Collaborating Centre for Infectious Disease Epidemiology and Control, School of Public Health, Li Ka Shing Faculty of Medicine, The University of Hong Kong, Hong Kong Special Administrative Region, 21 Sassoon Road, Pokfulam, Hong Kong, China; Hong Kong Centre of Occupational Medicine, Hong Kong Special Administrative Region, Hong Kong, China

**Keywords:** Acute respiratory infections, Health care workers, Sickness absence, Influenza, Pandemic

## Abstract

**Background:**

Acute respiratory infections (ARI) are a major cause of sickness absenteeism among health care workers (HCWs) and contribute significantly to overall productivity loss particularly during influenza epidemics. The purpose of this study is to quantify the increases in absenteeism during epidemics including the 2009 influenza A(H1N1)pdm09 pandemic.

**Methods:**

We analysed administrative data to determine patterns of sickness absence among HCWs in Hong Kong from January 2004 through December 2009, and used multivariable linear regression model to estimate the excess all-cause and ARI-related sickness absenteeism rates during influenza epidemics.

**Results:**

We found that influenza epidemics prior to the 2009 pandemic and during the 2009 pandemic were associated with 8.4 % (95 % CI: 5.6–11.2 %) and 57.7 % (95 % CI: 54.6–60.9 %) increases in overall sickness absence, and 26.5 % (95 % CI: 21.4–31.5 %) and 90.9 % (95 % CI: 85.2–96.6 %) increases in ARI-related sickness absence among HCWs in Hong Kong, respectively. Comparing different staff types, increases in overall absenteeism were highest among medical staff, during seasonal influenza epidemic periods (51.3 %, 95 % CI: 38.9–63.7 %) and the pandemic mitigation period (142.1 %, 95 % CI: 128.0–156.1 %).

**Conclusions:**

Influenza epidemics were associated with a substantial increase in sickness absence and productivity loss among HCWs in Hong Kong, and there was a much higher rate of absenteeism during the 2009 pandemic. These findings could inform better a more proactive workforce redistribution plans to allow for sufficient surge capacity in annual epidemics, and for pandemic preparedness.

## Background

Health care workers (HCWs) are an essential element for the efficient delivery of quality health services to a community. HCWs are exposed to many different health risks, including the risk of contracting various infectious diseases, in their occupational settings [1, 2]. Health problems in HCWs could negatively impact on workplace productivity, work efficiency, patient safety and quality of patient care [3]. Acute respiratory infections (ARIs), caused by a number of different pathogens, are the most common infectious causes of sickness absenteeism among workers [4]. Influenza virus infections, in particular, can contribute to significant productivity loss during annual epidemics and occasional pandemics [5].

For ARIs, besides an average 3–7 days of direct illness-related absence per episode [6], additional productivity loss can be indirectly incurred by the increased disease incidence in the community, particularly during an influenza epidemic. Parents of young children may have to be absent from work to look after their children who may have to stay home due to sickness or school closures [7]. The perceived risk of occupational exposure may also lead to unwillingness or refusal to go to work among a substantial proportion of the workforce [8–11]. These additional, indirect productivity losses can manifest as annual leave, unpaid leave, absence without official leave, or sick leave being labelling as any other illness.

Unanticipated manpower shortages among HCWs, due either to sickness, fear or other causes, may easily jeopardise the service delivery of a health care system during an influenza epidemic [12]. Service units or staff types lacking sufficient surge capacity are particularly vulnerable [13]. A proper understanding of the pattern of and factors affecting productivity loss in HCWs in relation to influenza epidemics/pandemics could help to inform contingency planning of human resource allocation and emergency preparedness [13].

The aim of our study is to describe the pattern of ARI-related sickness absence among HCWs in Hong Kong, to estimate the daily incidence of ARI-related sickness absence, and to quantify the impact on sickness absence associated with influenza epidemics during the study period including the influenza A(H1N1)pdm09 pandemic period in 2009.

## Methods

### Sources of data

The Hospital Authority (HA) in Hong Kong (HK) is responsible for delivering the majority of public health care services in HK. It is the second largest employer in HK with more than 60,000 staff across 42 public hospitals and more than 100 outpatient clinics. The Hong Kong West Cluster (HKWC) is one of the seven administrative clusters within HA, covering the Central, Western, and Southern districts of HK Island, consisting of one acute tertiary hospital and six convalescent hospitals together with general and specialist outpatient clinics of different specialities in each of these hospitals, and covering a population of around 500,000 persons, and employs around 7200 HCWs. The HKWC has the most developed occupational medicine service, and is the only cluster having a comprehensive sickness absence management service programme with components for continuous monitoring, early intervention, management and rehabilitation.

HA employees who took sick leaves were required to report their absence to their supervisors, which were in turn reported to the corporate human resources department for compilation. Each reporting form was accompanied with sickness absence certificate bearing the medical diagnosis issued by the attending doctors. This sick leave policy is unified organization-wide and applicable to all staff types and over the study period including the pandemic. We obtained a complete dataset recording all reported episodes of sickness absence in the HKWC from January 1, 2004 to December 31, 2009. Data records for each sickness absence episode included the diagnosis, duration of the sick leave, and staff type.

Data on the monthly numbers of staff stratified by staff types (medical/ nursing/ allied-health/ management/ supporting staff) over the study period were also obtained and used to estimate the number of full-time-equivalent (FTE) staff in each month. These five staff types comprise 9, 36, 10, 44 and 1 % of the total workforce respectively. Allied-health staff includes occupational therapists, radiographers, social workers and laboratory technicians; management staff includes hospital administrators, accounting officers, operational managers and clerical officers; and supporting staff includes workers who help the clinical staff and have frequent patient contacts, such as healthcare assistants, ward attendants and porters. The number of FTE staff were then linearly interpolated to obtained the daily number of FTE staff for all days in the corresponding month as the denominator for daily absence rate estimation. All-cause sickness absences were defined as absence from work for any sickness. ARI-related sickness absences were defined as those absence episodes labelled with ‘influenza’, ‘upper respiratory infection’ or ‘common cold’ as the diagnosis or reason for absence. We included both types of absenteeism in our study because ARI-related absence is a more specific indicator of the illness-related productivity loss caused directly by an acute infection, while all-cause sickness absence may be a better indicator of the overall impact on productivity loss related to the increased influenza activity during different periods, by reflecting also some other sickness absence taken not directly due to an acute infection.

### Ethics statement

Ethical approval was obtained for this study from the HA HKWC and University of Hong Kong institutional review board. All data were anonymised and no individual staff could be identified by the investigators.

### Statistical analysis

The number of reported sickness absences was aggregated for each calendar day for both all-cause and ARI-related sickness absences. The daily all-cause absence rates on each day were calculated by dividing the daily aggregated number of all-cause sickness absence by the interpolated daily number of FTE staff. Daily ARI-related absence rates were calculated by dividing the daily aggregated number of ARI-related sickness absence by the same daily number of FTE staff. The crude rates for the whole workforce as well as stratified rate by staff types were calculated.

For the pre-pandemic period (January 1, 2004 through 30 April 2009), each week was classified as either within a non-epidemic period or an epidemic period according to prevailing influenza activity in individual weeks. Taking reference from a previous local study, epidemic periods were defined as periods of two or more consecutive weeks in which at least 4 % of the annual number of virologically confirmed influenza diagnoses were recorded, according to the data on weekly positive influenza isolation rate from the virological reference laboratory in the HKWC [14]. The pandemic period was sub-divided into two sequential periods for the analysis, namely the containment period (May 1, 2009 – June 10, 2009) starting from the date that the World Health Organization issued a global alert, and the mitigation period (June 11, 2009 – December 31, 2009) starting from the occurrence of the first untraceable local case of A(H1N1)pdm09 in HK through to the end of the study period [15]. Mean daily all-cause and ARI-related absence rates observed among different staff types during the four periods were also analysed and compared.

In order to take into account other factors that can affect sickness absence among HCWs at different times, a multivariable linear regression model was used to model daily sickness absence rates among all staff and each staff types during the study period. Day-of-the-week effects, seasonal effects, long-holiday effects, and four different periods (non-epidemic/epidemic/containment and mitigation) as proxies of the effect of different influenza activity in the community were included in this multivariable model. The absolute excess sickness absence rate (% difference) was calculated for the epidemic, containment and mitigation periods with reference to the non-epidemic period. The relative excess in sickness absence rate (% increase) was also calculated by dividing the absolute excess by the corresponding mean rate in each period. All analyses were performed using R version 3.1.2 (R Foundation for Statistical Computing, Vienna, Austria).

## Results

During the 6-year study period, there were a total of 78743 episodes of sickness absence recorded, with a mean duration of 2.3 days (range: 0.5 days to 92 days). Among these, 27419 (34.8 %) episodes were classified as ARI-related, with each episode ranging from a period of 0.5-15 days and an average of 1.39 days. From the monthly manpower statistics, there was a mean number of 6880 (ranged from 6515 to 7454) HCWs working in the HKWC over the study period, of which around 9 % were medical staff, 37 % were nursing staff, 10 % were allied health staff, and 1 % administrative and 43 % supporting staff. Therefore the average staff time lost to absence was 82.8 person-days for all-cause absences, and 17.4 person-days for ARI-related absences.

**Fig. 1:**
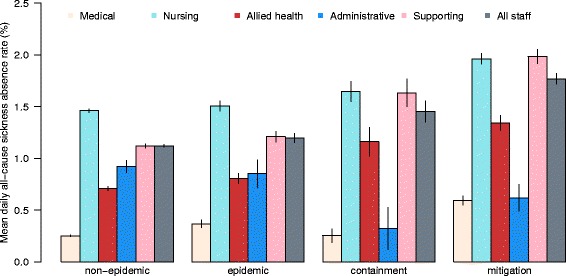
Mean daily all-cause sickness absence rates (and 95 % CI) by staff types over the study period

**Fig. 2:**
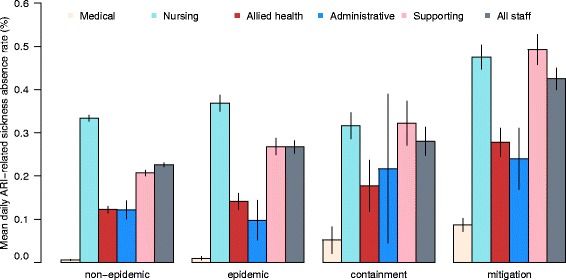
Mean daily ARI-related sickness absence rates (and 95 % CI) by staff types over the study period

ARI-related sickness absence accounted for an average of 20.5 and 23.2 % of the total number of daily absences during the pre-pandemic period and the 2009 influenza pandemic period respectively. Figs. 1 and 2 show the mean daily all-cause and ARI-related sickness absence rates for different staff types during the four periods, including non-epidemic and epidemic periods in the pre-pandemic period, and containment and mitigation phases of the pandemic period.

During non-epidemic periods, the overall mean daily all-cause and ARI-related absence rates were 1.12 and 0.23 % respectively. Nursing and supporting staffs had higher absence rates, with 1.46 and 1.12 % respectively for all-cause and 0.33 and 0.21 % respectively for ARI-related daily sickness absences. Medical staff had the lowest mean daily absence rate of 0.25 and 0.06 % respectively for all-cause and ARI-related absences during non-epidemic periods (Table 1). These differences in absence rates between staff types were statistically significant with p < 0.001, and agreed well with the similar finding of higher absence rates among nursing and supporting staff and lower rate among medical staffs from the general sickness absence audit done by HA for all public HCWs in HK in 2007 [16].

**Table 1:** Mean all-cause and ARI-related daily sickness absence rate among different staff types during different periods

All-cause sickness absence rate (%, SD)					
Staff type	Pre-pandemic period^a^		Pandemic period^b^		All periods
	Non-epidemic	Epidemic^c^	Containment^d^	Mitigation^e^	
Medical	0.25 (0.22)	0.37 (0.32)	0.25 (0.22)	0.59 (0.33)	0.30 (0.27)
Nursing	1.46 (0.37)	1.51 (0.43)	1.65 (0.33)	1.96 (0.37)	1.52 (0.41)
Allied Health	0.71 (0.41)	0.80 (0.43)	1.16 (0.47)	1.34 (0.53)	0.79 (0.47)
Administrative	0.92 (1.24)	0.85 (1.17)	0.32 (0.69)	0.62 (0.94)	0.87 (1.20)
Supporting	1.12 (0.41)	1.21 (0.45)	1.63 (0.45)	1.98 (0.51)	1.22 (0.50)
Overall	1.12 (0.32)	1.20 (0.38)	1.45 (0.35)	1.77 (0.48)	1.20 (0.39)
ARI-related sickness absence rate (%, SD)					
Staff type	Pre-pandemic period		Pandemic period		All periods
	Non-epidemic	Epidemic	Containment	Mitigation	
Medical	0.006 (0.029)	0.009 (0.038)	0.052 (0.105)	0.087 (0.111)	0.015 (0.053)
Nursing	0.33 (0.15)	0.37 (0.16)	0.32 (0.10)	0.48 (0.20)	0.35 (0.16)
Allied Health	0.12 (0.16)	0.14 (0.16)	0.18 (0.20)	0.28 (0.24)	0.14 (0.17)
Administrative	0.12 (0.44)	0.10 (0.40)	0.22 (0.58)	0.24 (0.51)	0.13 (0.45)
Supporting	0.21 (0.14)	0.27 (0.17)	0.32 (0.17)	0.49 (0.25)	0.24 (0.18)
Overall	0.23 (0.11)	0.27 (0.13)	0.28 (0.11)	0.42 (0.18)	0.25 (0.13)

ARI, acute respiratory infections

^a^Pre-pandemic period: from January 1, 2004 through April 30, 2009

^b^Pandemic period: from May 1, 2009 to December 31, 2009

^c^Epidemic periods: any two or more consecutive weeks with ≥4 % of the annual number of positive influenza isolation from the virological reference laboratory in the Hong Kong West Cluster [14]

^d^Containment period: from May 1, 2009 to June 10, 2009, starting from the date that the WHO issued a global alert till the occurrence of the first untraceable local case of A(H1N1)pdm09 in Hong Kong

^e^Mitigation period: from June 11, 2009 to December 31, 2009, starting from the occurrence of the first untraceable local case of A(H1N1)pdm09 in Hong Kong through to the end of the study period [15]

During the other three periods with increased influenza activities (i.e. the epidemic, containment, and mitigation periods), both overall daily absence rates increased in a stepwise manner (from 1.12 to 1.20, 1.45 and 1.77 for all-cause, and from 0.23 to 0.27, 0.28 and 0.42 for ARI-related), commensurate with the influenza activities in the community in different periods (Figs. 1 and 2, and Table 1). The differential daily absence rates between staff types and ranks (highest among nursing and supporting staff and lowest among medical staff) during the non-epidemic period were maintained during all the other three periods.

Using the linear regression model, the overall mean daily all-cause sickness absence rate increased from the baseline figure of 1.12 % in non-epidemic periods by an absolute amount of 0.09, 0.39, 0.65 % respectively during the three periods (epidemic, containment, and mitigation) (Table 2). Compared with the baseline amount of absence sickness, these figures represented a relative increase of 8.4, 35.0 and 57.7 % respectively. For all staff types besides administrative staff, the mean daily all-cause absence rates generally increased over the three periods, with the largest relative excess in the pandemic mitigation period. Figures for the pandemic containment period were unstable with wide confidence intervals due to the small amount of data in this very short period. In comparison to other staff types, the relative increase was highest for medical staff by 51.3 % (95 % CI: 38.9 %, 63.7 %) during epidemic period and 142.1 % (95 % CI: 128.0 %, 156.1 %) during the pandemic mitigation period. For administrative staff, there was a relative decrease in the absence rate during the pandemic period, although these estimates had wide confidence intervals due to the small size of this staff group.

**Table 2:** The absolute and relative change in mean daily all-cause sickness absence rates by periods^a^

Absolute change in mean daily all-cause sickness absence rate (%)			
Staff type	Epidemic period^b^	Containment period^c^	Mitigation period^d^
Medical	0.13 (0.10, 0.16)	0.03 (−0.05, 0.10)	0.36 (0.32, 0.39)
Nursing	0.07 (0.03, 0.10)	0.30 (0.20, 0.39)	0.48 (0.43, 0.52)
Allied Health	0.13 (0.09, 0.18)	0.44 (0.33, 0.54)	0.67 (0.62, 0.72)
Management	−0.08 (−0.23, 0.07)	−0.88 (−1.24, −0.53)	−0.21 (−0.38, −0.04)
Supporting	0.11 (0.06, 0.15)	0.57 (0.47, 0.67)	0.87 (0.82, 0.92)
Overall	0.09 (0.06, 0.13)	0.39 (0.32, 0.46)	0.65 (0.61, 0.68)
Relative change in mean daily all-cause sickness absence rate (%)			
Staff type	Epidemic period	Containment period	Mitigation period
Medical	51.3 (38.9, 63.7)	10.2 (−18.7, 39.1)	142.1 (128.0, 156.1)
Nursing	4.5 (1.8, 7.1)	20.2 (14.0, 26.4)	32.7 (29.7, 35.7)
Allied Health	19.3 (13.2, 25.4)	61.4 (47.0, 75.7)	94.5 (87.6, 101.5)
Management	−9.0 (−25.3, 7.4)	−96.3 (−134.6, −58.0)	−22.6 (−41.2, −4.0)
Supporting	8.6 (4.3, 12.9)	51.6 (41.5, 61.7)	77.6 (72.6, 82.5)
Overall	8.4 (5.6, 11.2)	35.0 (28.6, 41.4)	57.7 (54.6, 60.9)

^a^Regression model adjusted for day-of-the-week, holiday effect and seasonality. Positive figures represent an excess, negatives figures represent a decrease in sickness absence rate in the different periods, comparing to the baseline non-epidemic period

^b^Epidemic periods: any two or more consecutive weeks with ≥4 % of the annual number of positive influenza isolation from the virological reference laboratory in the Hong Kong West Cluster [14]

^c^Containment period: from May 1, 2009 to June 10, 2009, starting from the date that the WHO issued a global alert till the occurrence of the first untraceable local case of A(H1N1)pdm09 in Hong Kong

^d^Mitigation period: from June 11, 2009 to December 31, 2009, starting from the occurrence of the first untraceable local case of A(H1N1)pdm09 in Hong Kong through to the end of the study period [15]

Using the multivariable linear regression model, we estimated that the overall mean daily ARI-related sickness absence rate increased from the baseline figure of 0.23 % by an absolute amount of 0.06, 0.10 and 0.21 % during the three periods, representing a relative increase of 26.5, 43.2, and 90.9 % respectively (Table 3). For all staff types besides administrative staff, the mean daily ARI-related absence rates generally increased over the three periods, with the largest relative excess in the pandemic mitigation period. During epidemic period, the relative increase was highest among supporting staffs by 39.0 % (95 % CI 31.1 %, 46.9 %) and medical staff by 28.7 %, though the increase for medical staff was not significant (95 % CI −83.6 %, 141.0 %). In pandemic period, the relative increases of sickness absence among medical staff were almost 10 folds higher than those of other staff groups, of 7 times and 15 times respectively for the containment and mitigation periods. Figures were unstable with wide confidence intervals for administrative staff in the pandemic containment period.

**Table 3:** The absolute and relative change in mean daily ARI-related sickness absence rates by periods^a^

Absolute change in mean daily ARI-related sickness absence rate (%)			
Staff type	Epidemic period^b^	Containment period^c^	Mitigation period^d^
Medical	0.001 (−0.005, 0.008)	0.042 (0.028, 0.057)	0.082 (0.075, 0.089)
Nursing	0.06 (0.04, 0.08)	0.05 (0.01, 0.09)	0.15 (0.13, 0.17)
Allied Health	0.03 (0.01, 0.05)	0.06 (0.02, 0.11)	0.16 (0.14, 0.18)
Management	−0.004 (−0.061, 0.054)	0.118 (−0.017, 0.253)	0.117 (0.052, 0.183)
Supporting	0.08 (0.06, 0.10)	0.16 (0.12, 0.20)	0.29 (0.28, 0.31)
Overall	0.06 (0.05, 0.07)	0.10 (0.07, 0.12)	0.21 (0.19, 0.22)
Relative change in mean daily ARI-related sickness absence rate (%)			
Staff type	Epidemic period	Containment period	Mitigation period
Medical	28.7 (−83.6, 141.0)	771.2 (509.1, 1033.4)	1496.8 (1369.4, 1624.2)
Nursing	18.2 (13.1, 23.4)	14.5 (2.4, 26.5)	44.0 (38.1, 49.9)
Allied Health	21.1 (5.2, 37.1)	51.2 (14.1, 88.4)	132.5 (114.5, 150.6)
Management	−2.9 (−50.3, 44.5)	97.0 (−13.8, 207.7)	96.3 (42.5, 150.1)
Supporting	39.0 (31.1, 46.9)	76.9 (58.5, 95.4)	142.1 (133.1, 151.0)
Overall	26.5 (21.4, 31.5)	43.2 (31.4, 54.9)	90.9 (85.2, 96.6)

ARI, acute respiratory infections

^a^Regression model adjusted for day-of-the-week, holiday effect and seasonality. Positive figures represent an excess, negatives figures represent a decrease in sickness absence rate in the different periods, comparing to the baseline non-epidemic period/

^b^Epidemic periods: any two or more consecutive weeks with ≥4 % of the annual number of positive influenza isolation from the virological reference laboratory in the Hong Kong West Cluster [14]

^c^Containment period: from May 1, 2009 to June 10, 2009, starting from the date that the WHO issued a global alert till the occurrence of the first untraceable local case of A(H1N1)pdm09 in Hong Kong

^d^Mitigation period: from June 11, 2009 to December 31, 2009, starting from the occurrence of the first untraceable local case of A(H1N1)pdm09 in Hong Kong through to the end of the study period [15]

## Discussion

Our findings demonstrate the considerable impact that influenza epidemics and pandemics can have on HCWs’ productivity and the efficiency of health care delivery. In our study ARI-related absence was a major contributor (20 %) to all sickness absence, and the excess ARI-related absence observed in HK during epidemic and pandemic periods (26.5 % and 90.9 % respectively) reflects clearly the burden of influenza epidemics. The excess in all-cause absence, however, could be a better indicator of the potential surge impact as it reflects also additional illness-related absences that were not coded as ARI-associated, and other absence not directly caused by acute infections. For instance, part of the observed HCW productivity loss during the pandemic period may have been associated with the need of parents to stay home to care for their children during the prolonged school closures in June and July 2009, which could not be reflected from the sick leave statistics. The relative excesses of 8.4 % and 57.7 % during influenza epidemic and pandemic periods respectively indicated considerable productivity losses to the health care system.

**Fig. 3:**
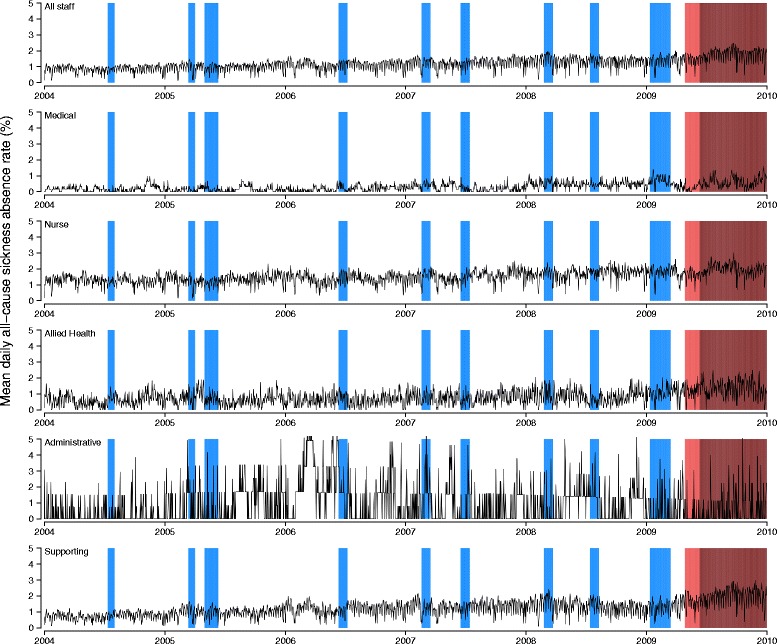
Mean daily all-cause sickness absence rates by staff types, 2004–2009. Shaded areas represent the epidemic (*blue*), containment (*pink*) and mitigation (*brown*) periods

The modest excess of all-cause absences (relative excess of 8.4 %) observed during influenza epidemic periods may be due to the more frequent and prolonged epidemics in subtropical HK [17] compared to temperate locations which typically only experience influenza epidemics in the winter, thus spreading the impact out over a longer period. In contrast, a study in Winnipeg found a 70 % increase in HCW absenteeism during the 1980–81 epidemic of influenza A/Bangkok 79, comparing to the corresponding period of the subsequent non-epidemic year [18]. General medical or paediatric nursing staff were most severely affected [18], similar to the observation of more absence among nurses in our study. Another similar study in Nottingham, however, failed to demonstrate any impact on staff absence during influenza epidemic seasons of 1993–94 and 1996–97 [19]. While local factors can vary in different geographical and temporal settings, the discrepancy may simply reflect seasonal variability in epidemic intensity captured by the very short study periods. Our estimation of the 0.09 % absolute and 8.4 % relative excess in all-cause daily sickness absence rates, and the 0.06 % absolute and 26.5 % relative excess in ARI-related daily sickness absence rates should be a more robust estimate being based on 6 years' data capturing 10 influenza epidemics (Fig. 3). As the uptake of seasonal influenza vaccination remained persistently poor among local HCWs in recent years (20–25 %) [20], possible options to improve uptake, including the employment of incentive or mandatory HCW vaccination could be considered.

Most of the current literature on productivity loss during a pandemic comes from hypothetical scenario-based surveys in a variety of hospital and community settings in different countries [8, 21–31]. The proportion of HCWs unwilling to work in the event of an influenza pandemic ranged from 16 to 85 %, indicating wide variability in potential absenteeism rates in different settings. The only survey of this type done in HK reported that 76.9 % of community nurses were unwilling to work in a pandemic [29], which agreed well with the estimate (83 % unwillingness) from a study on home HCWs in the United States [27], reflecting the high risk and poor level of protection in the community/home settings. Predictions from these surveys, however, contrasted greatly with our observed 1.77 % overall mean daily all-cause sickness absence rate among hospital-based HCWs during the pandemic mitigation period in HK. This was much lower than most estimates reported from previous hypothetical studies, and one reason for this is the generally lower severity of the A(H1N1)pdm09 virus compared to the severity profile of pandemic viruses that were anticipated in many scenario-based studies.

Nevertheless, the 15-fold higher excess in absenteeism among medical staff indicates a substantial impact of the pandemic on effective service delivery, particularly in units with limited surge capacity. Although our data did not allow for stratified analysis by unit or department because of the lack of corresponding stratified manpower data, overseas studies suggested that emergency department and critical care units caring for either adult or paediatric patients were among the most vulnerable units to manpower shortages because of their huge service need and limited surge capacity [31–33].

After the 2009 pandemic, several studies attempted to quantify its actual impact on sickness absence among general workers. A study in Norway reported an increase in overall absence rate (150 %), influenza-diagnosed sick leaves (73 %), GP-certified (130 %) and parental care work absence (400 %) captured by two employee registries in 2009–10 [34]. A study in Canada modelled data from labour force survey and estimated no change in absenteeism rates (13 % vs. 12 % per year) but longer duration of time-off (25 h vs. 14 h) due to the pandemic compared to a typical influenza epidemic [35]. A study in Brazil reported significantly increased ILI-related sick leaves among more than 11000 HCWs during the pandemic period from August to October 2009 compared with the same period in 2008 (884 vs. 96, p < 0.001), with a peak absence rate of 3 % of the workforce. Similar to our findings, nursing staff had the highest sickness absence rates [30]. The absolute (6.8 %) and relative excess (9 times higher) of this overall ILI-related sick leave were about one order of magnitude higher than our corresponding figures (0.21 and 90.9 % higher, respectively).

Our study has a few potential limitations. First, we may have underestimated the importance of ARI as a cause of absenteeism if some diagnoses were incorrectly coded on the sickness absence forms. Second, because of a lack of data we could not do stratified analysis by unit or department, although such an analysis could have given more insight into the consequences on absenteeism rates of heterogeneity in potential occupational exposures to influenza. Third, we selected one of the seven clusters of the HA in HK for our study, and we did not have data from the other clusters to confirm whether our results could be generalized to the rest of HK. Nevertheless, our data should be sufficiently representative as the distribution of staff types and other demographic characteristics were broadly similar to the territory-wide HA figures [36]. Finally, while all-cause sickness absence data may serve to reflect a portion of work absence indirectly related to ARI, we do not have suitable data to study other work absence burdens (unpaid annual leave, unpaid leave, absence without official leave potentially due to taking care of sick child or relative) and presenteeism, which may also contribute to the added productivity loss during epidemic/pandemic times.

## Conclusions

Our study demonstrated that influenza epidemics are associated with a substantial increase in sickness absence and productivity loss among HCWs in HK. Our findings highlight the importance of effective sickness absence surveillance and management with a proactive workforce redistribution plan to allow for sufficient surge capacity in annual epidemics. Increasing the uptake of influenza vaccination among local HCWs should be a priority to reduce influenza-associated absenteeism.
